# The Bioactivated Interfacial Behavior of the Fluoridated Hydroxyapatite-Coated Mg-Zn Alloy in Cell Culture Environments

**DOI:** 10.1155/2011/192671

**Published:** 2011-11-17

**Authors:** Jianan Li, Lei Cao, Yang Song, Shaoxiang Zhang, Changli Zhao, Fan Zhang, Xiaonong Zhang

**Affiliations:** ^1^State Key Laboratory of Metal Matrix Composites, School of Materials Science and Engineering, Shanghai Jiao Tong University, Shanghai 200240, China; ^2^Shanghai Key Laboratory of Orthopaedic Implant, Department of Orthopaedics, Shanghai Ninth People's Hospital, Shanghai Jiao Tong University School of Medicine, Shanghai 200011, China

## Abstract

A partially fluorine substituted hydroxyapatite- (FHA-) coated Mg-Zn alloy was prepared to investigate the interfacial behavior of degradable Mg-based biomaterials with degradable bioactive coatings in a cell culture environment. Peaks from the results of X-ray diffraction (XRD) were characterized and compared before and after cell culture. It was found that Ca-P, including poorly crystalline ion-substituted Ca-deficient HA (CDHA), was formed in greater amounts on the interface of coated samples compared with the uncoated ones. A thermodynamic mechanism for Ca-P formation on biodegradable Mg alloys in a cell culture environment is proposed. Combined with improved cell calcification, the-FHA coated Mg alloys have the ability to promote CDHA formation, as expected thermodynamically. It is suggested that the specific cell culture environment and the bone-like FHA coatings together facilitate the observed behavior. Moreover, cell culture environment probably increased the biomineralization to a detectable level by affecting the kinetics of apatite formation.

## 1. Introduction

The biological formation of minerals by living organisms is commonly called biomineralization [1–3]. One of the important compositions is Ca-P-rich layer. In the use of orthopaedic implants made by Mg-based biodegradable biomaterials, these formations may not only consist of minerals from the cell side but also precipitation from surrounding solutions. Most studies in biodegradable magnesium have paid attention to the behaviors of the cells and surrounding tissues because of the existence of biomaterials. But little effort has been devoted to the changes of the biomaterials' interface after being used in a cell culture environment. The purpose of the research presented here is trying to characterize the behavior of the interface in a cell culture environment. Meanwhile, the behavior was compared with that in the modified simulated body fluid (m-SBF) [4].

Aiming at that and based on our previous studies [5, 6], the FHA-coated (nominal formula Ca_5_(PO_4_)_3_(OH)_1-x_F_x_) Mg-Zn alloy which showed better corrosion resistance than the electrodeposited DCPD (nominal formula CaHPO_4_ *·*2H_2_O) and HA (nominal formula Ca_10_(PO_4_)_6_(OH)_2_) were selected and prepared as the test samples.

## 2. Materials and Methods

### 2.1. Coating Preparation

The electrolyte for fabricating the FHA coating on Mg alloys by electrodeposition process contained 0.042 M Ca(NO_3_)_4_·4H_2_O, 0.1 M NaNO_3_, 0.025 M NH_4_H_2_PO_4_, 6 vol% H_2_O_2_, and 1–1.2 × 10^−3^ M NaF. It was firstly prepared according to our previous studies [5, 7]. Extruded Mg-Zn alloy is used as the substrate. The chemical composition can be seen in our previous study [8]. The current density of electrodeposition was about 0.5 mA/cm^2^ and the processing time was 2-3 hours. The thickness of the coating is about 2–8 *μ*m [5, 6]. The samples were subsequently sterilized by ethylene oxide (EO) gas. For the sake of reducing the remaining EO, all the specimens including the sterilized uncoated alloys were placed into the laminar flow cabinet for about 2 weeks before cell culture experiments. The characterization was taken and described in our previous studies [5, 6].

### 2.2. The Behavior in the Cell Culture Environment

About 5 × 10^3^ human bone marrow stromal cells (hBMSCs) were seeded on each substrate with and without coatings. The samples with cells were normally cultured, and the osteogenic *α*-MEM was used and changed every 3 days. The cells were supplied by the Department of Orthopaedic Surgery in the Ninth People's Hospital of Shanghai according to the reported method [9]. The cell culture medium (*α*-MEM, Invitrogen, Carlsbad, CA) contained 10% fetal bovine serum (FBS, PAA) and was modified to be osteogenic one by adding 50 *μ*g/mL L-ascorbic acid, 10 mM glycerophosphate, and 100 nM dexamethasone to induce the osteoblastic differentiation. After 14 days, the samples were washed by PBS and fixed in the 95% ethanol. The mineral nodules were labeled by incubating the samples in culture medium that contains tetracycline for about 30 minutes [10]. Then, the areas of biomineralization were observed by Axio Fluorescence Microscope (machine Carl Zeiss, Scope A1 for BioMed) and calculated by Image-Pro Plus software (USA). For the statistical analysis, three replicates of samples in each group of materials were tested. All data are shown as means ± standard errors. The areas of calcium depositions were evaluated using six photographs for every sample. Statistical differences were defined as *P* < 0.05 in one-way ANOVA analysis. The behavior of the interface was evaluated by analyzing phase changes before and after cell culture. The cells were detached from the samples using trypsin-EDTA (Invitrogen; Carlsbad, CA). Afterwards, XRD (machine Rigaku, D/MAX255) data were collected from 20° to 60° (2*θ*) using CuK*α* radiation at a step of 0.02° with a scanning speed of 5°/min. The XRD patterns were compared with the curves detected in the previous m-SBF immersion test [4, 5]. Three replicates of samples in each group of materials were tested and typical curves were selected.

## 3. Results

**Figure 1:**
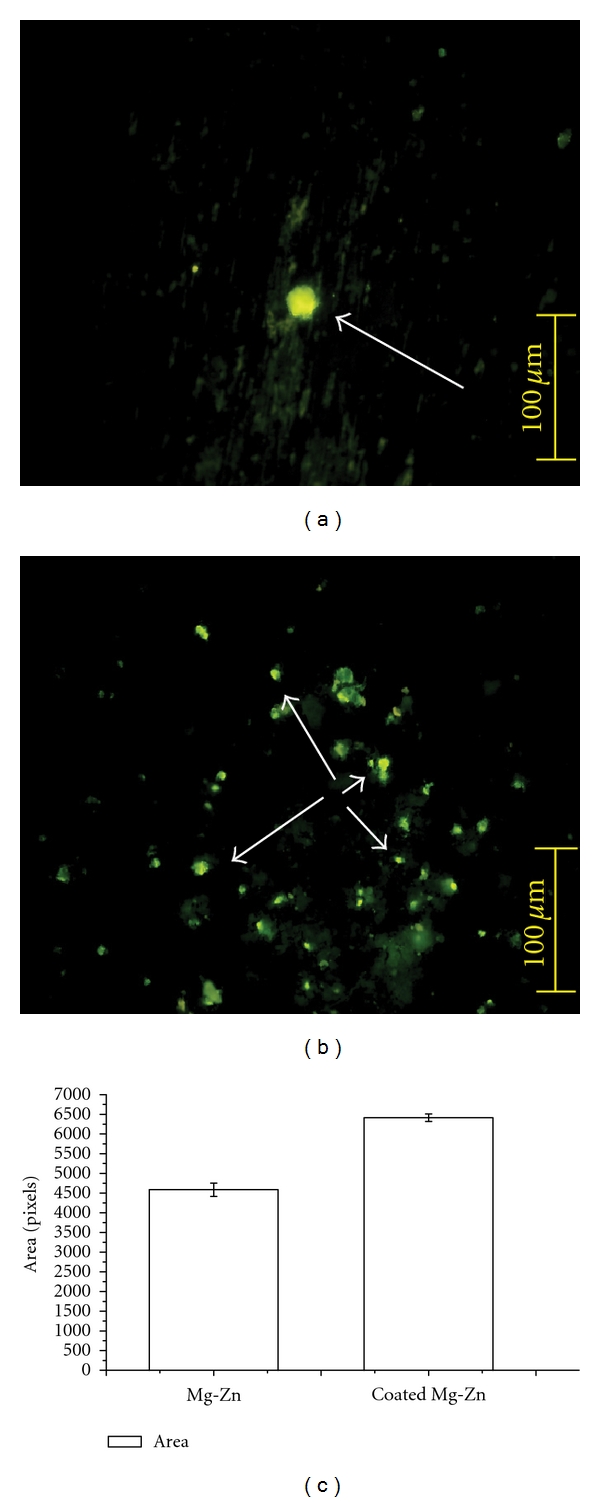
Typical calcification and calcium deposition (white arrows) fluorescence photographs after 14 days' culture in osteogenic medium and labeled in tetracycline. (a) The result on the Mg-Zn alloy. Scale bar = 100 *μ*m. (b) The result on the coated one. Scale bar = 100 *μ*m. (c) The calculated size of mineralization area of hBMSCs after 14 days of culture (mean ± SD,*P* < 0.05).

The ability of cells to form mineralized nodules is an important part of biomineralization for biomaterials.  Figure 1 reveals the representative morphology of the mineralized nodules on the 14th day. A small number of wider nodules are observed on the Mg-Zn alloy (Figure 1(a)) while a large number of smaller ones can be seen on the FHA-coated alloy (Figure 1(b)). Consequently, the entire stained mineralization area was bigger on the coated sample than that on the uncoated one, as shown in Figure 1(c). The quantitative difference has a statistical definition as *P* < 0.05 in one-way ANOVA analysis.

**Figure 2:**
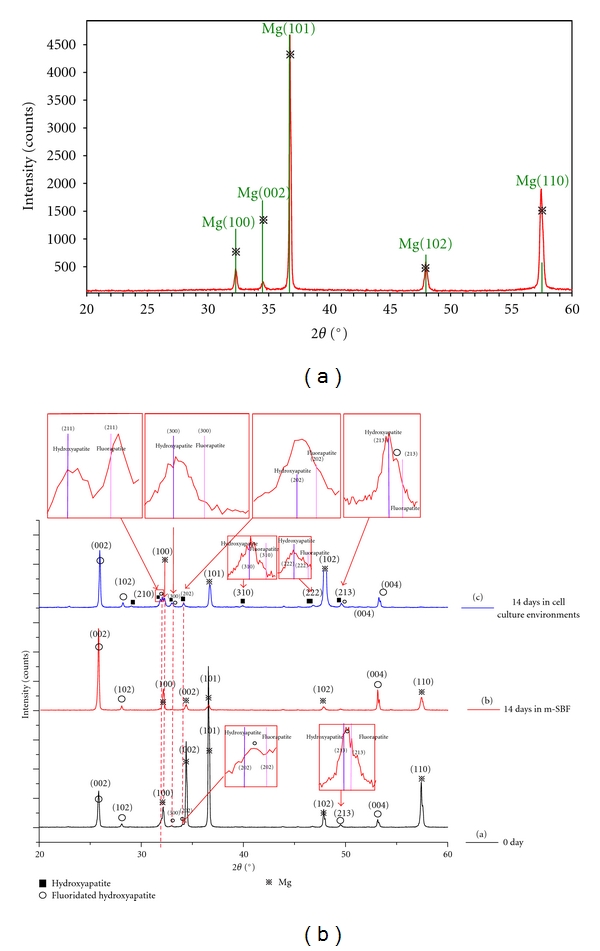
Typical XRD diffraction pattern. (a) The result on the Mg-Zn alloy after 14 days' culture and detaching the cells from the sample. (b) The result on the FHA-coated one. Curve-a presents the diffraction pattern of the FHA coatings right after the electrodeposition. Curve-b presents the pattern of the FHA coatings after being immersed in m-SBF for 14 days. Curve-c presents the diffraction pattern of the FHA coatings after 14 days' culture in cell culture environment and detaching the cells from the sample.

**Figure 3:**
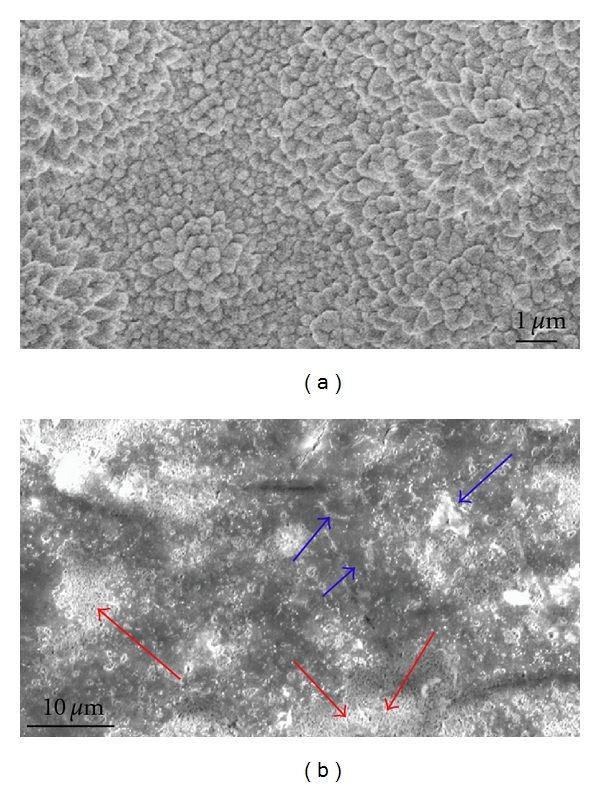
Typical SEM pictures of the coatings. (a) The picture before cell culture. Scale bar = 1 *μ*m. (b) The picture after cell culture and detaching the cells from the sample. Scale bar = 10 *μ*m. The red arrow indicated the remaining coating while the blue arrow indicated newly appeared layers or depositions.

As can been seen in Figure 2(a), there were no other peaks other than Mg after detaching the cells from the Mg-Zn alloy. As for the FHA-coated Mg-Zn alloy, the peaks were identified in Figure 2(b). There is no significant difference between curve-a and curve-b, except for the weaker peak of crystal plane (2 1 3) in curve-b. No distinct changes appeared after 14 days immersion in m-SBF [4], which is widely used to evaluate degradation of biodegradable materials. Conversely, it should be noticed that all the FHA peaks existed almost the same as can be seen in curve-c after 14 days in the cell culture environment. Importantly, major peaks belongs to HA which showed no signals in m-SBF immersion test came into sight in curve-c. Meanwhile, the peaks around 2*θ* = 32.2° which were mixed with the strong (1 0 0) peak of magnesium became more clear. Among those mixed peaks, major peaks of HA tagged (2 1 1), (3 0 0), and (2 1 3) appeared. Those peaks shifted around the same plane of fluorapatite. As a result, the interface after 14 days cell culture was composed of remaining FHA coating and newly appeared poorly crystalline CDHA as can be seen in Figure 3(b). The apparent differences between samples in m-SBF immersion test and cell culture environment may indicate the ability of cell culture environment in promoting the biomineralization level. The promotion ability showed up in the appearance of more and better crystallized Ca-P formation on the interface of biomaterials. Additionally, the FHA coating could also result in enhanced biomineralization in comparison with the bare Mg-Zn alloy.

## 4. Discussion

A number of investigators found that the corrosion rates of the same magnesium alloy obtained from different kinds of corrosion tests usually exhibit different corrosion rates [11, 12]. Proteins such as albumin have been demonstrated to form a corrosion-blocking layer on the magnesium alloys in the in vitro experiments [13, 14]. Meanwhile, the ions released from both the magnesium base and the interface can also work on the cell function, which can react on the degradation of the biomaterial in turn. Therefore, a major thrust of this paper is the changes at the interface of the biomaterials in cell culture environment, which is important in bone healing and fixation.

### 4.1. Effects Taken to the Cells' Mineralization

From the point view of cells, the results in Figure 1 demonstrate that all of the samples supported the hBMSCs' mineralization behavior. Permanent tetracycline labels could deposit with the bone mineral at the calcification front in the presence of active mineralization of new osteoid and thus in centers of new bone formation [10, 15–17]. As can be seen, greater numbers of nodules with smaller per-area appeared on the FHA-coated samples while less but wider ones on the Mg-Zn alloy. We have found that Mg-Zn alloy can conduct hBMSCs into differentiation stage more quickly than FHA-coated ones [6]. Those might cause the wider area, as shown in Figure 1(a). But at the same time, the higher alkaline environments which are not suitable for cell to live might lead to the smaller numbers on the Mg-Zn alloy. Consequently, the entire area of the stained mineralization on the coated samples was larger than that on the uncoated one. Thus, the FHA-coating can assist the biomineralization of hBMSCs compared to the Mg-Zn alloy without coating.

### 4.2. Behavior of the Interface in the Cell Culture Environment

When switching the focus to the behavior of the implant, it was found that the interfacial behavior of the FHA-coated alloy differed on account of the whole cell culture environment. In Mg research area, m-SBF is widely used in the in vitro immersion test because of its stability and similarity to blood plasma in terms of its ion concentrations [4], as can be seen in Table 1. However, the components of a cell culture environment vary from medium designed for different cell types and culture aims, even the same product with different catalog numbers. For example, *α*-MEM, D-MEM, and E-MEM for different cells, and also common medium compared with osteogenic ones. Besides considering all the different kinds of mediums above, there are significant differences between the components in *α*-MEM and m-SBF. According to Table 1, there are more additives essential for living cells in culture medium. Moreover, proteins like fetal bovine serum (FBS) and osteogenic additives were also contained in the culture environment in this study. All the additives as well as the ions from inorganic salts in the medium are not still. The concentration will change under the effect of cells absorption and secretion. That is why the cell culture medium should be changed every several days to make sure cells are alive. Because of the tight correlation between living cells and culture medium, they were treated as one factor, called whole cell culture environment in this study.

The detaching solution was trypsin-EDTA (Invitrogen; Carlsbad, CA) which can fully detach the cells and little of the corrosion products due to the complexing reaction between the released Mg^2+^and EDTA [18, 19]. Consequently, the XRD pattern is the structure of the interface itself. As for the uncoated Mg-Zn alloy, no other peaks except for Mg were detected in the pattern. Most of the corrosion products might be washed away due to the low precipitation-interface bonding by the detaching solution. Another possibility is that the depositions were amorphous. As a result, XRD could not detect the peaks. However, as for the FHA-coated Mg-Zn alloy, it should be noticed that more peaks belonging to CDHA appeared after cell culture. The mineral formation is better than that on the bare Mg-Zn alloy. After 14 days in cell culture environment, the interface was composed of remaining FHA coating and depositions including CDHA. The compositions were quite different from the interface left after 14 days' immersion in m-SBF.

### 4.3. Typicality in the Area of Biodegradable Mg Study

Precipitation from the medium is an important part of biomineralization in biodegradable biomaterial research. For each crystallization and precipitation process, the most important requirement is supersaturation, which is the driving force for nucleation to occur. According to the research carried out by Yamamoto and Hiromoto [12], the thermodynamic precipitation behavior in *α*-MEM is predicted and estimated as follows. The concentrations were selected from those in *α*-MEM, liquid production from Invitrogen in Table 1. The calculation was made based on the data at 25°C for the situation here at 37°C according to [20], because the later ones are not available. The estimated equilibrium concentration of PO_4_^3−^ is

(1) $$\left[{\text{P}{\text{O}}_{4}}^{3-}\right]=\frac{C_{\text{total}}\left({\text{P}{\text{O}}_{4}}^{3-}\right)K_{a1}K_{a2}K_{a3}}{\left\{{\left[H^{+}\right]}^{3}+{\left[H^{+}\right]}^{2}\;K_{a1}+\left[H^{+}\right]K_{a1}K_{a2}+K_{a1}K_{a2}K_{a3}\right\}},$$

where *K*_*a*1_, *K*_*a*2_, and *K*_*a*3_ are the dissociation constants of H_3_PO_4_, H_2_PO_4_^−^, and HPO_4_^2−^ as 6.92 × 10^−3^, 6.17 × 10^−8^, and 4.79 × 10^−13^ [12, 21]. *C*_total_ (PO_4_^3−^) in *α*-MEM is about 1.01 mM and when Mg alloys begin to degrade, the local pH value will increase rapidly which makes the [H^+^] strongly decrease. The equilibrium concentration [PO_4_^3−^] will be larger and at the same time the [OH^−^] will also increase. When the pH value of the solution is 7.4 at the beginning, the [H^+^] is about 3.98 × 10^−8^ M and the [OH^−^] is about 2.5 × 10^−7^ M. Then the equilibrium concentration of PO_4_^3−^ at the pH of 7.4 will be 7.39 × 10^−9^ M according to the equation above. The K_sp_ of stoichiometric HA is about 10^−117^(*K*_sp_(Ca_10_(PO_4_)_6_(OH)_2_) ≈ 10^−117^) [20, 22–24]. Since there is 1.8 × 10^−3^ M Ca^2+^in *α*-MEM, the ion-product *Q*_sp_(Ca_10_(PO4)_6_(OH)_2_) = [Ca^2+^  ]^10^[PO_4_^3−^]^6^[OH^−^]^2^ = [1.8  ×  10^−3^]^10^ × [7.39  ×  10^−9^]^6^ × [2.5  ×  10^−7^]^2^≈3.6 × 10^−90^. Thus HA will precipitate thermodynamically according to *Q*_sp_ > *K*_sp_, from the beginning of the test. When the precipitation happens, CDHA would form instead of pure HA because of the existence of other inorganic ions in the solutions. Similarly, the nucleation opportunities for calcium phosphate or other magnesium phosphate can also be calculated in the same way especially when the pH of the medium increases [12]. When Mg alloy and the coating begin to degrade, all the concentration of Ca^2+^, PO_4_^3−^, and OH^−^ will increase. The *Q*_sp_ will be larger according to the previous equations. Consequently, CDHA could precipitate more easily during the degradation of Mg alloy according to the thermodynamical calculation. The appearance of major peaks of HA in the 14-day results confirmed this suggestion.

Moreover, abundant Ca^2+^ and PO_4_^3−^ can be used in ions mobilization and take part in the cell calcification, which plays another important role in biomineralization besides precipitation [25, 26]. Combined with the better cell calcification, the FHA-coated Mg alloys have the ability to have CDHA formation. And the formation of another Ca-P-rich layer can be transformed into stable CDHA on the interface of the coated samples, as can be seen in Figure 2(b). The formation of apatite is both thermodynamically expected and kinetically favored. The whole cell culture environment may enhance the ability mostly in a kinetic way, which needs more detailed studies to clarify.

## 5. Conclusion

A kind of Ca-deficient, partially F substituted hydroxyapatite- (FHA-) coating was prepared by the electrodeposition process on the Mg-Zn alloy. The interface behavior in the cell culture environment was characterized by XRD. In contrast with artificial biological environments like m-SBF, more and rapidly formed stable Ca-P formations including poorly crystalline CDHA were induced on the interface of the coated samples. The specific cell culture environment and the bone-like coatings together facilitate the appearance of this phenomenon. Additionally, cell culture environment promoted the biomineralization behavior to a detectable level.

## Figures and Tables

**Table 1:** The concentration of components in different solutions.

Concentration/mM						
		Blood plasma [4]		m-SBF [4]	*α*-MEM, Invitrogen, liquid	*α*-MEM, Invitrogen, powder
		Total	Dissociated			

Ion from inorganic salts	Na^+^	142.0	142.0	142.0	144.44	118.25
	K^+^	5.0	5.0	5.0	5.33	5.33
	Mg^+^	1.5	1.0	1.5	0.814	0.814
	Ca^2+^	2.5	1.3	2.5	1.8	1.8
	Cl^−^	103.0	103.0	103.0	126.17	126.17
	HCO_3_^−^	27.0	27.0	10.0	26.19	**0**
	HPO_4_^2−^	1.0	1.0	1.0	**0**	**0**
	SO_4_^2−^	0.5	0.5	0.5	0.814	0.814
	H_2_PO_4_^−^	**0**	**0**	**0**	1.01	1.01

Amino acids		nd	nd	**0**	21 kinds	21 kinds

Vitamins		nd	nd	**0**	11 kinds	11 kinds

Ribonucleosides		nd	nd	**0**	Various	Various

Deoxyribonucleosides		nd	nd	**0**	Various	Various

Other components				HEPES	Various	Various

HEPES: 4-(2-hydroxyerhyl) piperazine-1-erhanesulfonic acid, C_8_H_18_N_2_O_4_S.

nd: no data available.

Various: components vary from products that have different catalog numbers.
